# Differences in Extended-Spectrum Beta-Lactamase Producing *Escherichia coli* Virulence Factor Genes in the Baltic Sea Region

**DOI:** 10.1155/2014/427254

**Published:** 2014-08-28

**Authors:** Jana Lillo, Kristiine Pai, Arta Balode, Mariia Makarova, Kristi Huik, Siiri Kõljalg, Marina Ivanova, Lidia Kaftyreva, Jolanta Miciuleviciene, Paul Naaber, Kristel Parv, Anastasia Pavelkovich, Tiiu Rööp, Karolin Toompere, Ludmila Suzhaeva, Epp Sepp

**Affiliations:** ^1^Department of Microbiology, University of Tartu, Ravila Street 19, 50411 Tartu, Estonia; ^2^Riga Stradiņš University, 16 Dzirciema Street, LV-1007 Riga, Latvia; ^3^St. Petersburg Pasteur Institute, Ul Mira 14, St. Petersburg 197101, Russia; ^4^East-Tallinn Central Hospital, Ravi 18, 10138 Tallinn, Estonia; ^5^Vilnius City Clinical Hospital, Antakalnio Street 57, LT-10007 Vilnius, Lithuania; ^6^Quattromed HTI Laboratories, Väike-Paala 1, 11415 Tallinn, Estonia

## Abstract

The aim of this study was to compare the prevalence of different virulence factor (VF) genes in extended-spectrum beta-lactamase (ESBL) producing *Escherichia coli* strains isolated from the Baltic Sea region. A total of 432 strains of phenotypically ESBL positive *E. coli* were collected from 20 institutions located in Estonia, Latvia, Lithuania, and the region of St. Petersburg in Russia from January to May 2012 and analyzed for phylogenetic group and prevalence of 23 VF genes. The strains were collected from clinical material (urine, blood, wound, and respiratory tract). Bacterial isolates were compared according to phylogenetic group, clinical material, and geographical origin. Most of the VF genes were concentrated within phylogenetic group B2 and/or D. When comparing strains isolated from different countries, it was found that strains originating from Estonia and Latvia belonged mainly to group B2 and strains from Lithuania and Russia mainly to groups B2 and D. The P-fimbrial adhesin gene *papEF* was more prevalent in Russian strains, colicin gene *cvaC* in Lithuanian strains, and capsular gene *kpsMTII* in Latvian strains; serum resistant gene *traT* was less prevalent in Estonian strains. The regional differences of VF genes remained statistically significant after taking into account the phylogenetic distribution in the countries.

## 1. Introduction

*Escherichia coli* strains, which are important to humans, can be classified into 3 groups: commensal strains, intestinal pathogenic (enteric or diarrheagenic) strains, and extraintestinal pathogenic* E. coli* (ExPEC) strains [1]. ExPEC strains can cause infections in almost every organ or anatomical site, typically urinary tract infections, neonatal meningitis, intra-abdominal infections, pneumonia, soft-tissue infections, and bacteremia [1, 2].

Pathogenic* E. coli* clones have acquired specific virulence factors (VF), which confer an increased ability to adapt to new niches and allow them to cause a broad spectrum of diseases [3]. ExPEC isolates have functionally similar VF profiles and clonal background, and they are distinct from commensal and intestinal pathogenic* E. coli* strains [4]. VF-s of ExPEC include different adhesins, toxins, capsules, siderophores, invasins, and antibiotic resistance. These VF-s contribute to colonization and invasion into host tissues, avoidance to immune responses, and antimicrobial drugs and acquiring nutrients from the host [5, 6]. The management of infections caused by* E. coli* is complicated due to the increasing resistance to antibiotics. Extended-spectrum *β*-lactamase (ESBL) production is a common mechanism of resistance to third-generation cephalosporins in* E. coli*, associated with the frequent use of *β*-lactam antibiotics in treatment of serious* E. coli* infections [5].

*E. coli* strains can be divided into four main phylogenetic groups: A, B1, B2, and D [7]. The strains causing extraintestinal infections belonging preferentially to group B2 and to a lesser extent to group D. Commensal strains largely belong to groups A and B1 [8, 9]. However, the distribution of phylogenetic groups may vary in different geographic regions. It depends on the climatic zone and environmental factors [10].

The aim of this study was to compare the phylogenetic distribution and prevalence of different VF-s in extended-spectrum *β*-lactamase producing extraintestinal* E. coli* strains isolated from the Baltic Sea region.

Some information presented in this paper was previously demonstrated at 23rd ECCMID held on 27–30, April, in Berlin, Germany [11].

## 2. Materials and Methods

### 2.1. Strains

A total of 423 strains of phenotypically ESBL positive* Escherichia coli* were investigated. All consecutive ESBL positive strains were collected from patients from 20 institutions located in Estonia (*n* = 5), Latvia (*n* = 4), Lithuania (*n* = 3), and the region of St. Petersburg in Russia (further referred to as Russian strains) (*n* = 8) from January to May 2012. The strains were isolated from different clinical materials (Table 1), identified as* E. coli* by Matrix Assisted Laser Desorption/Ionization Time of Flight instrument (MALDI-TOF; Bruker Daltonik GmbH, Germany) and ESBL production was confirmed by ROSCO ESBL kit (Rosco Diagnostica A/S, Denmark) [12].

### 2.2. Phylogenetic Analysis

All bacterial strains were assigned to one of the four main* E. coli* phylogenetic groups (A, B1, B2, and D) according to PCR-based method published by Clermont et al. [7].

### 2.3. Virulence Genotyping

All bacterial isolates were screened for 23 VF genes coding for adhesins (*papAH*,* papC, papEF*,* papGI*,* papGII*,* papGIII*,* fimH*,* sfa/focDE*,* focG, nfaE*, and* bmaE*), toxins (*hlyA*,* cvaC*, and* cdtB*), capsule synthesis (*kpsMTII, kpsMTIII*,* kpsMT K1*, and* rfc*,), siderophore systems (*fyuA* and* iutA*), invasin (*ibeA*), pathogenicity island (*PAI*) marker of highly virulent uropathogenic* E. coli* strain CFT073, which is used as a marker for uropathogenic PAIs [13], and serum resistance (*traT*).

In order to determine 23 VF genes, four previously described multiplex-PCR primer sets were used: (i)* PAI*,* papAH*,* fimH*,* kpsMTIII*,* papEF*, and* ibeA*; (ii)* fyuA*,* bmaE*,* sfa/focDE*,* iutA*,* papGIII*, and* kpsMT K1*; (iii)* hlyA*,* rfc*,* nfaE*,* papGI*,* kpsMTII*, and* papC*; (iv)* cvaC*,* cdtB*,* focG*,* traT*, and* papGII* [14].

Total bacterial DNA was purified using PureLink Pro96 Genomic DNA Kit (Invitrogen, USA). Amplification was done in 25 *μ*L mixtures containing 2 *μ*L (200 ng) of template DNA, 1x HotStart PCR buffer (Thermo Scientific, USA), 0.2 *μ*M of 4 dNTPs, 0.6 *μ*M of each primer, 2.5 mM of MgCl_2_, and 1U HotStart DNA polymerase (Thermo Scientific, USA).

PCR conditions were as follows: 4 min at 94°C, followed by 30 cycles of 30 s at 94°C, 30 s at 55°C for primer set (i) and 30 s at 63°C for primer sets (ii)–(iv), 1.30 min at 72°C, and finally 15 min incubation at 72°C. The PCR products were analyzed by electrophoresis (150 V, 400 mA for 1.5 h) in 2% agarose gel prepared in 1xTris-acetate-EDTA (TAE) buffer, stained with ethidium bromide (0.5 *μ*g/mL).

For each of the detected genes, one PCR product was sequenced and compared with* E. coli* DNA sequences on NCBI BLAST (http://blast.ncbi.nlm.nih.gov/) in order to control whether primers bind on correct region in bacterial DNA. Controlled DNA samples were further used as positive controls.

### 2.4. Statistical Analysis

Fisher's exact test was used to compare the prevalence of the 23 individual VF genes between strains isolated from different countries, materials, and strains belonging to different phylogenetic groups. Comparisons of VF scores were assessed using Mann-Whitney *U* test. VF score was calculated as sum of virulence genes detected, adjusted for multiple detection of the* pap*,* sfa*,* foc*, and* kpsMTII* operon (*papAH*,* papEF, papC*,* kpsMT K1*, and* focG* were not taken into account). In order to assess the impact of phylogenetic group and country of origin to the VF score while controlling for the possible contribution from the other variable in the model, the mutually adjusted odds ratios with 95% confidence intervals were calculated using fractional logit models [15]. The models were fitted for (1) all VF genes (adjusted for multiple detection of the* pap*,* sfa*,* foc*, and* kpsMTII* operons), (2) adhesin genes (adjusted for multiple detection of* pap*,* sfa*, and* foc* operons), (3) capsule synthesis genes (adjusted for multiple detection of* kpsMTII* operon), (4) toxin genes, and (5) genes coding for siderophores. The group least likely to carry VF gene was chosen as reference group. The criterion for significance was taken as *P* < 0.05.

## 3. Results

### 3.1. VF Genes

All of the 423 extraintestinal ESBL-producing* E. coli* isolates contained at least one of the VF genes studied. In total, 26 (6%) strains contained 1–3 VF genes, 208 (49%) strains 4–6 VF genes, 164 (39%) strains 7–9 VF genes, and 24 (6%) strains 10–13 VF genes. No strain contained more than 13 out of 23 studied VF genes.

The prevalence of VF genes ranged from 0% (*bmaE* and* papGI*) to 94% (*fimH*) (Table 2).

### 3.2. Comparison of VF Genes in Strains according to Phylogenetic Group

Among the 423* E. coli* isolates, 26 (6%) strains belonged to phylogenetic group A, 17 (4%) strains to group B1, 300 (71%) strains to group B2, and 80 (19%) strains to group D.

The different phylogenetic groups exhibited disparate VF scores (mean ± standard deviation): group A 3.9 ± 1.6, group B1 4.8 ± 1.5, group B2 6.0 ± 1.3, and group D 5.3 ± 1.3. Strains belonging to group B2 were found to carry significantly more VF genes than strains belonging to A, B1, and D (*P* < 0.001; *P* < 0.001; *P* < 0.001). Strains belonging to group A were found to carry significantly less (*P* < 0.001) VF genes than strains belonging to group D.

Most of the genes were found to be more prevalent in groups B2 and/or D (Table 3). In group B2, capsular gene* kpsMTII*, siderophore gene* fyuA* and pathogenicity island marker* PAI* were more prevalent and at the same time toxin gene* cvaC* was less prevalent as compared to the other three groups. P-fimbrial adhesin gene* papEF* was more prevalent in group D when compared to the other groups.

### 3.3. Comparison of VF Genes in Strains according to Clinical Material

When comparing* E. coli* strains according to clinical material, no statistical differences in phylogenetic distribution were found. There were differences in prevalence of 3 VF genes: P-fimbrial adhesin gene* papGII* was found more frequently in strains isolated from respiratory tract than in strains from urine (34.2% versus 14.3%;  *P* = 0.004), and capsular gene* kpsMTII* was also found more frequently in strains isolated from respiratory tract than in strains from blood (60.5% versus 29.6%;  *P* = 0.02). In strains isolated from wound, siderophore gene* iutA* was found more frequently than in strains isolated from urine and blood (90.2% versus 77.8% and 70.4%;  *P* = 0.01 and *P* = 0.02).

### 3.4. Comparison of VF Genes in Strains according to Geographical Origin

The ESBL-producing* E. coli* strains in Estonia and Latvia belonged mostly to phylogroup B2 and in Lithuania and Russia to groups B2 and D (Table 4).

There were differences in prevalence of 15 VF genes. Compared to the other 3 countries, P-fimbrial adhesin gene* papEF* was more prevalent in Russian strains, toxin gene* cvcC* was more prevalent in Lithuanian strains, capsular gene* kpsMTII* was more prevalent in Latvian strains, and serum resistance gene* traT* was less prevalent in Estonian strains (Table 5).

### 3.5. What Affects the Existence of VF Genes: Phylogenetic Group or Origin of Strains?

In fractional logit models, some regional differences remained statistically significant after taking into account the phylogenetic distribution in the countries (Table 6). The odds ratio (OR) of all appointed VF genes was higher in strains isolated from Latvia (OR 1.1, 95% CI: 1.0–1.2) and the OR of adhesin genes was higher in strains isolated from Russia (OR 1.2, 95% CI: 1.0–1.3) compared to strains isolated from Estonia. The capsule synthesis genes were least represented in Russian strains; strains isolated from Latvia (OR 1.8, 95% CI: 1.4–2.3) and Lithuania (OR 1.4, 95% CI 1.0–1.9) were carrying significantly more capsule synthesis genes compared to Russian strains. Siderophores were more than twice as likely to be represented in Russian (OR 2.7, 95% CI 1.4–5.4) than in Lithuanian strains.

The VF scores were also associated with the phylogenetic group after adjusting for country of origin. Strains belonging to the phylogenetic group A were least likely to carry VF genes (Table 6). The OR for all VF genes and adhesin genes was significantly higher for strains belonging to phylogenetic groups B1 (OR 1.3, 95% CI 1.0–1.7 and OR 1.4, 95% CI 1.0–1.9), B2 (OR 1.8, 95% CI 1.5–2.2 and OR 1.8, 95% CI 1.3–2.3), and D (OR 1.5, 95% CI 1.2–1.9 and OR 1.6, 95% CI 1.2–2.1). In phylogenetic group B2, the OR of capsule synthesis genes (OR 1.7; 95% Cl: 1.2–2.5) and siderophores (OR 3.5; 95% Cl: 1.7–7.1) was higher than in group A. In phylogenetic groups B2 and D, siderophore genes were twice as likely (OR 3.5, 95% Cl 1.7–7.1 and OR 2.4, 95%  Cl 1.1–5.3) as in groups B1 or A.

## 4. Discussion

In this study, we characterized the collection of 423 extraintestinal phenotypically ESBL positive* E. coli* strains with respect to phylogenetic groups and 23 VF genes. To our knowledge, the present study is the first to assess the phylogenetic distribution and prevalence of VF genes within the* E. coli* strains isolated from the Baltic Sea region.

ESBL positive* E. coli* strains belonged mostly to phylogenetic groups B2 and D, which contained more VF genes compared to groups A and B1.* E. coli* strains isolated from Estonia and Latvia belonged mostly to phylogenetic group B2 and strains isolated from Lithuania and Russia mainly to groups B2 and D. Phylogenetic group and country of origin were associated with prevalence of VF genes, whereas clinical materials from which the strains were isolated were not.

Most of the studied ESBL positive* E. coli* strains belonged to phylogenetic group B2 (71%) and group D was the second most common phylogenetic group (19%). Our results support some previous results obtained with ESBL positive* E. coli* strains isolated from different clinical material (blood, urine, wound, and sputum) [16, 17] but do not match the observations made by Branger et al. and Rodrígues-Baño et al., where group B2 was represented, respectively, only in 36.4% and in 15.4% of the ESBL-producing strains isolated from different clinical materials and strains causing bloodstream infections [18, 19]. They explain the scarce prevalence of group B2 in their studies with higher antibiotic resistance of strains belonging to other phylogroups than B2. Also, the occurrence of resistance encoding integrons has been found more frequent in* E. coli* strains of phylogenetic group B2 compared to non-B2 strains [20]. Strains belonging to phylogroups A, B1, and D express significantly less VF genes and invade more commonly compromised hosts; hence, less VF-s would be required to cause infections in such patients. Antibiotic resistance gives such strains an advantage to cause infections; previous antibiotic treatment was common, which would have been selected for ESBL* E. coli* [18, 19].

The distribution of VF genes in different phylogenetic groups was not even. As reported previously, most of the genes, in which case differences in distribution were observed, were more prevalent in phylogenetic groups B2 and/or D [19, 21–23].* E. coli* strains belonging to group B2 showed the highest virulence score, which is concordant with previous studies [19, 21, 23]. In group B2, siderophore gene* fyuA*, capsular gene* kpsMTII*, and pathogenicity island marker* PAI* were more prevalent and colicin gene* cvaC* was less prevalent than in other phylogenetic groups. Carattoli et al. found pathogenicity island in all ESBL positive* E. coli* strains, whereas the great majority of the strains belonged to phylogenetic group B2 [24]. In strains belonging to phylogenetic group D, P-fimbrial adhesin gene* papEF* was more prevalent than in strains from other groups.

We found the virulence gene profile to be significantly associated with the geographical origin of the strain. There were differences in prevalence of 15 out of 23 VF studied genes among* E. coli* strains isolated from different countries. An explanation for this may be because of the different phylogenetic distribution of* E. coli* strains originating from different countries in the Baltic Sea region; as in the current study and previous studies by other authors, the distribution of VF genes has been found to differ between phylogroups [16, 17, 19].* E. coli* strains originating from Estonia and Latvia showed a similar distribution to phylogenetic groups: more strains belonged to group B2 and less to groups B1 and D in comparison with Lithuanian and Russian strains. Strains isolated from Lithuania and Russia belonged to groups D and B2.

Some differences in prevalence of VF genes seemed to be explainable by differences among phylogenetic distribution: P-fimbrial adhesin (*pap*) genes occurred more frequently in Russian strains. The difference in prevalence of* pap-*genes could be explained by differences in the phylogenetic distribution of strains, because half of the Russian* E. coli* strains belonged to group D and we found that* pap*-genes are associated with group D. However, after adjusting for phylogenetic distribution, we found that Russian strains showed higher statistical probability of containing adhesin genes, which indicates that there could be also other explanations for higher prevalence of* pap-*genes in Russian strains. The same could be said about capsular gene* kpsMTII*, which was found to be associated with group B2 and occurred more frequently in Latvian strains, where prevalence of group B2 was the highest. But again we found that the odds ratio of capsular genes in Latvian strains was the highest, so higher prevalence of* kpsMTII* might not be explainable by differences in phylogenetic distribution. Although comparing prevalence of siderophore genes* fyuA* and* iutA* did not show higher proportion of these genes in Russian strains, we found that when differences in phylogenetic distribution were taken into account, siderophore genes were more than twice as likely to be represented in Russian strains.

To our knowledge, there are only a few studies that compare the prevalence of virulence genes in ExPEC strains isolated from different countries [25, 26]. Grude et al. compared* E. coli* strains that were isolated from patients with bacteriuria from Russia and Norway. They found differences in phylogenetic distribution and virulence gene profile: Russian isolates belonged mainly to phylogenetic group A, while phylogroups B2 and D were predominant among the Norwegian isolates. Norwegian isolates also had a significantly higher number of virulence genes compared to isolates from Russia [25]. Differences observed could be due to geographic and climatic factors, as it has been found that they play an important role in structuring* E. coli* population worldwide, including also commensal populations [10, 27]. As the geographic region observed in the current study is small (Baltic countries and region of St. Petersburg in Russia) and countries involved have a similar climate, then differences found are probably caused by other factors.

Our study revealed no significant relatedness between infection site and phylogenetic distribution or virulence gene profile of studied ESBL producing* E. coli* strains. This indicates the importance of host factors in the process of infection development. We only found significant differences in prevalence of 3 VF genes (*papGII, kpsMTII,* and* iutA*) and no significant differences in phylogenetic distribution. Lee et al. had similar results: they found that distribution of phylogenetic groups was similar between isolates from different clinical materials (blood and urine), but in contrast to the current study, they observed differences in prevalence of other genes (*fyuA*,* traT*, and* PAI)*, which were more prevalent in strains isolated from blood than in strains from urine [23]. There is not enough data about VF genes of ExPEC strains isolated from other infections than urinary tract infections, bacteremia, and neonatal meningitis, but some authors have described that strains which colonize respiratory tract and other body locations could be similar to strains isolated from blood and urine. This could explain the scarceness of differences among strains isolated from different clinical materials [28, 29].

In conclusion, our study indicates that the prevalence of particular virulence factors in extraintestinal extended-spectrum beta-lactamase producing* E. coli* strains is associated with phylogenetic group and geographical origin of strains rather than infection site. The regional differences of VF genes in ESBL positive* E. coli* strains remained statistically significant after taking into account the phylogenetic distribution in the countries.

## Figures and Tables

**Table 1:** Number of ESBL producing *E. coli* strains isolated from different countries and materials.

Origin of strains (*n*)	Estonia (*n* = 149)	Latvia (*n* = 112)	Lithuania (*n* = 35)	Russia (*n* = 127)
Urine (*n* = 266)	110 (74%)	52 (46%)	19 (54%)	85 (67%)
Blood (*n* = 27)	7 (5%)	8 (7%)	7 (20%)	5 (4%)
Wound (*n* = 92)	23 (15%)	37 (33%)	7 (20%)	25 (20%)
Respiratory tract (*n* = 38)	9 (6%)	15 (13%)	2 (6%)	12 (9%)

**Table 2:** The prevalence of 23 VF genes among 423 extraintestinal ESBL producing *E. coli* strains, isolated from the Baltic Sea region.

VF	Gene	Prevalence (% of total)
Adhesins	*bmaE *	0 (0)
	*fimH *	399 (94)
	*focG *	5 (1)
	*nfaE *	2 (0.5)
	*papAH *	65 (15)
	*papC *	101 (24)
	*papEF *	67 (16)
	*papGI *	0 (0)
	*papGII *	71 (17)
	*papGIII *	8 (2)
	*sfa/focDE *	45 (11)

Toxins	*hlyA *	68 (16)
	*cvaC *	49 (12)
	*cdtB *	2 (0.5)

Capsule	*kpsMTIII *	251 (59)
	*kpsMT K1 *	22 (5)
	*rfc *	5 (1)
	*kpsMTII *	204 (48)

Siderophores	*fyuA *	352 (83)
	*iutA *	341 (81)

Invasin	*ibeA *	37 (9)

Pathogenicity island	*PAI *	215 (51)

Serum resistance	*traT *	364 (86)

**Table 3:** VF-s exhibiting significant prevalence differences according to phylogenetic distribution among 423 extraintestinal ESBL producing *E. coli* strains, isolated from Baltic Sea region.

VF	Gene	Number of isolates (% of total)				*P*
		A^a^ (*n* = 26)	B1^b^ (*n* = 17)	B2^c^ (*n* = 300)	D^d^ (*n* = 80)	
Adhesins	*papAH *	3 (12)	0 (0)	44 (15)	19 (24)	0.02^bd^
	*papC *	4 (15)	0 (0)	73 (24)	24 (30)	<0.02^bc,bd^
	*papEF *	1 (4)	0 (0)	44 (15)	22 (27)	<0.02^ad,bd,cd^
	*papGII *	1 (4)	1 (6)	50 (17)	19 (24)	0.02^ad^
	*fimH *	19 (73)	16 (94)	288 (96)	76 (95)	<0.001^ac,ad^

Toxins	*hlyA *	0 (0)	0 (0)	53 (18)	15 (19)	<0.02^ac,ad^
	*cvaC *	7 (27)	5 (29)	23 (8)	14 (17)	0.005^ac^; <0.02^bc,cd^

Capsule	*kpsMTII *	2 (8)	3 (18)	174 (58)	25 (31)	<0.001^ac,bc,cd^; 0.02^ad^

Siderophore	*fyuA *	15 (58)	11 (65)	263 (88)	63 (79)	<0.001^ac^; <0.05^ad,bc,cd^
	*iutA *	17 (65)	13 (76)	242 (81)	69 (86)	0.04^ad^

Pathogenicity island	*PAI *	3 (11)	5 (29)	186 (62)	21 (26)	<0.001^ac,cd^ 0.01^bc^

Serum resistance	*traT *	18 (69)	14 (82)	262 (87)	70 (87)	<0.04^ac,ad^

^a,b,c,d^Indicate strains isolated from different phylogenetic groups.

**Table 4:** Phylogenetic distribution of 423 extraintestinal ESBL producing *E. coli* strains isolated from different countries.

Phylogenetic group	Number of isolates (% of total)				*P*
	Estonia^a^ (*n* = 149)	Latvia^b^ (*n* = 112)	Lithuania^c^ (*n* = 35)	Russia^d^ (*n* = 127)	
A	11 (7)	3 (3)	3 (9)	9 (7)	—

B1	3 (2)	2 (2)	1 (3)	11 (9)	<0.02^ad,bd^

B2	121 (81)	100 (89)	22 (63)	57 (45)	0.02^ac^; <0.001^ad,bc,bd^

D	14 (9)	7 (6)	9 (26)	50 (39)	0.02^ac^; ≤0.003^ad,bc,bd^

^a,b,c,d^Indicate strains isolated from different countries.

**Table 5:** VF-s exhibiting significant prevalence differences according to geographical origin among 423 ESBL producing *E. coli* strains.

VF	Gene	Number of isolates (% of total)				*P*
		Estonia^a^ (*n* = 149)	Latvia^b^ (*n* = 112)	Lithuania^c^ (*n* = 35)	Russia^d^ (*n* = 127)	
Adhesins	*papAH *	14 (9)	8 (7)	5 (14)	38 (30)	≤0.001^ad,bd^
	*papEF *	13 (9)	10 (9)	3 (9)	41 (32)	≤0.001^ad,cd^; 0.005^bd^
	*papC *	20 (13)	29 (26)	6 (17)	46 (36)	≤0.05^ab,cd^; 0.001^ad^
	*papGII *	3 (2)	24 (21)	4 (11)	40 (31)	≤0.02^ac,cd^; ≤0.001^ab,ad^
	*sfa/focDE *	28 (19)	9 (8)	5 (14)	3 (2)	≤0.02^ab,cd^; 0.001^ad^
	*focG *	2 (1)	1 (1)	2 (6)	0 (0)	0.05^cd^

Toxins	*hlyA *	24 (16)	12 (11)	4 (11)	28 (22)	0.02^bd^
	*cvaC *	18 (12)	13 (12)	10 (29)	8 (6)	≤0.03^ac,bc^; ≤0.001^cd^

Capsule	*kpsMTII *	78 (52)	77 (69)	9 (26)	40 (31)	≤0.008^ab,ac,ad^; ≤0.001^bc,bd^
	*kpsMTIII *	81 (54)	80 (71)	27 (77)	63 (50)	0.01^ac^; ≤0.007^ab,bd,cd^
	*rfc *	0 (0)	0 (0)	1 (3)	4 (3)	0.04^ad^

Siderophores	*fyuA *	125 (84)	90 (80)	25 (71)	112 (88)	0.03^cd^
	*iutA *	106 (71)	101 (90)	28 (74)	108 (85)	0.02^bc^; ≤0.001^ab,ad^

Invasin	*ibeA *	27 (18)	3 (3)	4 (11)	3 (2)	0.04^cd^; ≤0.001^ab,ad^

Serum resistance	*traT *	110 (74)	110 (98)	33 (94)	111 (87)	≤0.007^ac,ad,bd^ 0.001^ab^

^a,b,c,d^Indicate strains isolated from different countries.

**Table 6:** Estimated odds ratios (OR) and 95% confidence intervals (CI) for carrying of all VF genes and genes belonging to specific VF groups.

VF genes	OR (95% Cl)							
	Countries				Phylogenetic groups			
	Estonia	Latvia	Lithuania	Russia	A	B1	B2	D
All VF genes	1	**1.1** **(1.0–1.2)**∗	1.1 (0.9–1.2)	1.1 (0.9–1.2)	1	**1.3** **(1.0–1.7)**∗	**1.8** **(1.5–2.2)**∗	**1.5** ** (1.2–1.9)**∗

Adhesins	1	1.0 (0.9–1.2)	1.1 (0.9–1.4)	**1.2** **(1.0–1.3)**∗	1	**1.4** **(1.0–1.9)**∗	**1.8** **(1.3–2.3)**∗	**1.6** **(1.2–2.1)**∗

Capsule synthesis genes	1.3 (0.9–1.7)	**1.8** **(1.4–2.3)**∗	**1.4** **(1.0–1.9)**∗	1	1	1.2 (0.6–2.2)	**1.7** **(1.2–2.5)**∗	1.4 (0.9–2.1)

Toxins	1.3 (0.8–2.2)	1	1.8 (0.9–3.6)	1.1 (0.6–2.0)	1.0 (0.5–2.1)	1.2 (0.5–2.8)	1	1.5 (0.9–2.3)

Siderophores	1.2 (0.6–2.2)	1.8 (0.9–3.7)	1	**2.7** **(1.4–5.4)**∗	1	1.2 (0.5–3.0)	**3.5** **(1.7–7.1)**∗	**2.4** **(1.1–5.3)**∗

*Statistically significant OR.
